# Barriers to Engaging in Blood Donation During the COVID‐19 Pandemic Among Nondonors and Lapsed Donors in a Chinese Community: A Critical Medical Anthropology Perspective

**DOI:** 10.1111/hex.70236

**Published:** 2025-04-02

**Authors:** Judy Yuen‐man Siu, Engle Angela Chan, Angus Siu‐cheong Li, Yik Mun Lee

**Affiliations:** ^1^ Department of Applied Social Sciences, Faculty of Health and Social Sciences The Hong Kong Polytechnic University Hong Kong People's Republic of China; ^2^ International Research Centre for the Advancement of Health Communication The Hong Kong Polytechnic University Hong Kong People's Republic of China; ^3^ Research Centre for SHARP Vision The Hong Kong Polytechnic University Hong Kong People's Republic of China; ^4^ School of Nursing, Faculty of Health and Social Sciences The Hong Kong Polytechnic University Hong Kong People's Republic of China; ^5^ Hong Kong Red Cross Blood Transfusion Service Hospital Authority Hong Kong People's Republic of China

**Keywords:** barriers, blood donation, COVID‐19, critical medical anthropology, lapsed donors, nondonors

## Abstract

**Background:**

The COVID‐19 pandemic has presented a major challenge to maintaining a stable blood supply. In Hong Kong, the percentage of eligible donors who donated blood dropped from 2.7% before the pandemic to 2.34% and 2% during the pandemic.

**Objective:**

This study explored barriers to blood donation among nondonors and lapsed donors during the COVID‐19 pandemic.

**Methods:**

A critical medical anthropology framework and a qualitative descriptive design were used. In‐depth semi‐structured interviews were conducted individually between February and July 2021 in Hong Kong with 80 adults aged 19–65 years who were nondonors or who had previously donated blood but had lapsed from doing so.

**Results:**

The participants who did not donate blood during the pandemic reported multiple reasons that arose during the pandemic and before it. The decision to not donate is sometimes the outcome of a social process established before the pandemic. Although institutional infection control and quarantine policies were most relevant for nondonation during the pandemic, policy and structural factors intertwined and created new social and cultural ideals that demotivated participants from donating blood. The difficult relationship between mainland China and Hong Kong as well as participants' unpleasant experiences with personnel in donor centres served as underlying barriers before the pandemic.

**Discussion:**

The decision not to donate during the pandemic cannot be explained by pandemic factors alone. Although the participants' sense of being a ‘good citizen’ arising from the new social norms developed in the pandemic at the intermediate level (quarantine policy) and the macro‐level social structure (collective responsibility) had affected their micro‐level perceptions (blood donation as unnecessary and risky and healthcare personnel as dangerous), their experiences at different social levels preceded the pandemic had played an important embedding role in reinforcing their nondonation during the pandemic.

**Conclusion:**

To enhance the motivation to donate blood among nondonors and lapsed donors, merely addressing the barriers arising from the pandemic is inadequate. Prepandemic factors should also be addressed.

**Patient or Public Contribution:**

The participants shared their experiences in the interviews. All participants had read and confirmed the content of their transcripts and referred more participants for this study.

## 1 Introduction

Infectious disease outbreaks affect people's intentions to donate blood [1], and the COVID‐19 pandemic endangered blood banks worldwide. Blood donation decreased during the pandemic by 25%–71% in different countries [2]. The number of blood donors in Zhejiang province in China dropped by 67%, and the success rate of donor recruitment dropped by 60% [3]. Hong Kong has faced similar challenges in maintaining a stable blood supply during the pandemic due to pressure from quarantine orders, social distancing policy, restriction and suspension of social activities, and the government's recommendation to work and study at home [4, 5].

Alerts for low blood reserve were issued several times during the pandemic [4], but such challenges had been a longstanding problem in Hong Kong even before the pandemic. The blood supply in Hong Kong is sustained entirely by voluntary and nonremunerated donations under the central management of the Hong Kong Red Cross Blood Transfusion Service (BTS) [6]. The BTS runs 11 donor centres in different districts and one mobile donation vehicle [6]. Nevertheless, in 2018 and 2019 (before the pandemic), there were 142205 and 146200 people had ever donated blood (2.7% of the donation‐eligible population) [7, 8]. During the pandemic, number of blood donors only counted for 121,740 (2.34%) in 2020, 121,222 in 2021 and then dropped further to 119,967 (2%) in 2022 [9, 10, 11]. In view of the high percentage of those who are eligible but choose not to donate, it is important to recruit nondonors and lapsed donors to donate blood [12], which has become even more pressing in light of the pandemic.

Haw et al. suggest it is important to investigate the response of nondonors to the COVID‐19 pandemic and their motivations to donate blood during that time to stabilise the blood supply during public health crises [13]. Commonly mentioned reasons for not donating during the pandemic include worries about becoming infected [3] and policies related to infection control and quarantine [14, 15]. In a study comparing blood donation intentions between prepandemic and the first pandemic phase in April to June 2020 in Germany, inactive donors' perceived ability to donate significantly dropped during the pandemic [16]. Furthermore, inactive donors feel less responsible and less morally obliged to donate during the pandemic [16]. However, these reasons do not completely explain the low donation rates. A survey in Hong Kong shows that 80% of respondents had not resumed blood donation after the heaviest wave of the pandemic [17]. Thus, even though the pandemic has passed, other reasons must be playing a role in the decision not to donate. This study, therefore, aims to investigate both the reasons related and unrelated to the pandemic. Understanding those reasons, including examination of factors not directly related to the pandemic, could assist health policymakers in designing more effective recruitment strategies for both nondonors and lapsed donors.

## 2 Methods

This study adopted a qualitative descriptive approach [18]. A critical medical anthropology (CMA) framework [19] was used to investigate the barriers to blood donation among nondonors and lapsed donors at the individual, micro, intermediate and macro levels. Semi‐structured in‐depth individual interviews were conducted with 80 adults aged 19–65 years who were living in Hong Kong during the study period of February to July 2021.

### 2.1 Theoretical Framework and Methodological Underpinning

**Figure 1:**
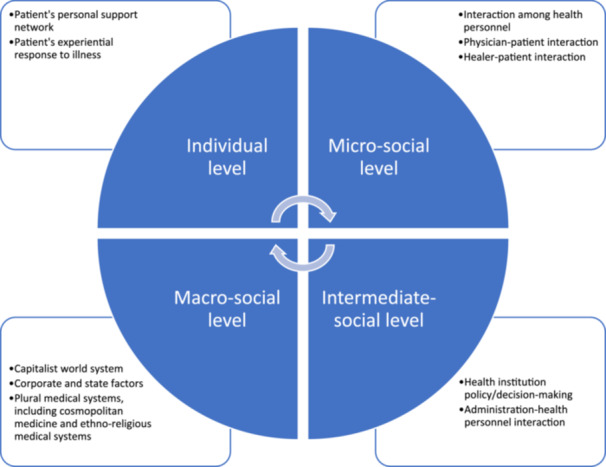
Concept map of critical medical anthropology framework adopted from Baer et al. [19].

Qualitative studies allow for a detailed understanding of an issue, including how the context impacts participants' experiences and perceptions [20]. Lambert and McKevitt advocate the value of anthropology in healthcare research as such an approach regards people's lay perceptions and actions as socially and culturally specific and valid within that frame [21]. This is important because narrowly framing an investigation in biomedical terms limits the ability to design interventions that are responsive to community needs [21]. A systematic review shows that the lay perspectives of health beliefs and experience in affecting donation‐decision processes for nondonors still remain understudied [22]. Studies in various cultural contexts have shown that blood donation in nonpandemic times can be influenced by factors such as religious beliefs [23, 24, 25, 26], social expectations [26, 27], family ties [28] and subjective trust in a healthcare system [29]. An ethnographic study in India indicates that the context of religious gift‐giving, sacrifice, caste, kinship, and nationalism can affect blood donation practice [30]. Whereas during pandemic times, personal perceptual factors, government institutional factors and social factors that developed from the new social norms are noted as factors in donating blood for active donors in a Chinese community [31]. Although the pandemic is shown to have less impact on active donors' motivation in Germany [16], it has a stronger impact on nondonors in Australia and Germany during pandemic times, with subjective norms and self‐efficacy affecting their intention to donate [16, 32]. Hence, across different social contexts, multilevel social and cultural factors were identified as relevant influences on blood donation during pandemic and nonpandemic times, which suggests the utility of taking a holistic approach to the study of this phenomenon. Anthropology, and specifically CMA, are therefore appropriate tools for understanding complex decision‐making processes around blood donation during the COVID‐19 pandemic. CMA investigates the interrelationship between people's micro‐level experience, the intermediate‐level of social organisation action and the macro‐level of social structure (Figure 1). Furthermore, it explicitly recognises connections among social groups to larger national and global societies [33]. This framework emphasises how power structures can affect people's health beliefs and behaviours, particularly in and through social relationships [34].

According to CMA, global, capitalist, political and economic forces can affect human relationships, social behaviours and collective experiences [19]. Further, factors at the individual, micro‐social, intermediate‐social and macro‐social levels are interlocking and can help to explain health behaviours [19]. At the individual level, personal factors and social networks can influence a person's individual donation behaviour. At the micro‐social level, the interaction between people and healthcare personnel can influence decisions to donate. At the intermediate‐social level, institutional policies of governments can affect people's decisions. Finally, at the macro‐social level, ethnocultural beliefs of a society and the political economy embedded in the global system can affect a person's decision.

Anthropological studies are important for placing objects of study in a social context; this frequently involves descriptive analysis [34]. A qualitative descriptive approach was used in this study to describe the perceptions and experiences of participants [18]. Qualitative description recognises the subjective nature of human experience, and the findings are presented in a way that directly reflects or closely resembles the terminology used by research participants [35]. It illuminates how people feel and experience things and the factors they think facilitate or hinder their behaviours [36]. Qualitative description is characterised by a lower level of interpretation and inference from researchers, so the researchers can stay closer to the data and the words used by informants [37]. The CMA framework adopted in this study is well‐suited to contextualise perceptions and experiences in the social and cultural context specific to a Chinese population.

### 2.2 Ethical Considerations

Ethics approval was obtained from the Human Subjects Ethics Subcommittee of The Hong Kong Polytechnic University before the start of the study (HSEARS20171013002). Written informed consent (for face‐to‐face interviews) or audio‐recorded informed consent (for online interviews) was obtained from the participants before data collection.

### 2.3 Data Collection

Study participants were selected by purposive and snowball sampling. The following criteria were used for the selection: age 18 to 65 years, never having donated blood (nondonors) or not having donated blood for 3 years after a prior donation made before 1 January 2018 in Hong Kong (lapsed donors), being born and educated in Hong Kong, residence in Hong Kong at the time of the study. Blood donors were considered as lapsed if they had not donated for 36 consecutive months at the time of recruitment following literature [38].

These sampling criteria ensured that the participants had been exposed to the social environment of Hong Kong over the long term. The eligible age for blood donation ranges from 16 to 75 years in Hong Kong [39]. However, those aged 16–17 years can only donate blood with parental consent, and those aged over 66 years are required to undergo annual health assessments and receive approval from the BTS [39]. Therefore, only those who were between 18 and 65 years of age during the study period were sampled to minimise the potential impact of parents and health conditions.

The participants were recruited in three ways. Some participants (*N* = 4) were undergraduate students who had responded to a survey conducted at a university about the perception of blood donation [40]. Others were recruited via materials posted in public areas at the same university (*N* = 6 nondonors and 2 lapsed donors). The remaining participants (30 non‐ and 38 lapsed donors) were recruited from the broader community, including from social service agencies and residents' associations in different districts, a doctor's fan page on Facebook (with 25,000+ followers during the study period), and referrals from prior participants. Informational recruitment posters indicating the eligibility criteria were provided to these organisations and the owner of the Facebook page after obtaining the consent from these agencies, associations and the doctor's Facebook for participant recruitment.

An open‐ended interview guide (Supporting Information: Appendix 1) was developed after referencing literature on blood‐donation perceptions and barriers [1, 25, 41, 42, 43, 44, 45]. Although the questions were designed to correspond to the four levels of the CMA framework to examine the factors, probing questions that are not in the interview guide were asked following the responses of each participant to dig into the participants' experiences. The participants were interviewed individually by the same interviewer, who had undergraduate and master's‐level education in sociology. The interviewer received intensive training and supervision from the first and second authors throughout the data‐collection process.

All interviews were conducted in Cantonese (the native language of the participants and the interviewer). Because the interviews were conducted during the COVID‐19 pandemic, the participants had the option of an online interview. Ultimately, four interviews were conducted face‐to‐face, whereas the remaining 76 interviews were conducted online. Each interview lasted 1–1.5 h, and the audio was recorded with the participant's consent. To compensate the participants for their time, each one was given a supermarket cash coupon worth HK$200 (approximately 25.64 USD) upon completion of the interview.

### 2.4 Data Analysis

After each interview, the interviewer documented the key points and impressions on each interview in an interview diary. The first and second authors also noted the key points and impressions when listening to the audio recordings of all interviews. The notes by the first three authors were compared and discussed throughout the data‐collection process. Interviews were transcribed verbatim, and the transcripts were examined line by line in the coding process. The first, second and third authors separately coded the interview transcripts using thematic analysis, according to Braun and Clarke [46]. The codes, categories and themes identified along with supporting interview quotes were documented in a coding table [47].

As this study follows the CMA framework, identified codes, categories and themes were consolidated into four social levels for analysis. Other data not following the CMA framework was also coded inductively and documented in the coding table. Data saturation is defined as data redundancy with no new themes or codes emerge from further interviews [48], which was achieved at the 31st interview for nondonors and at the 34th interview for lapsed donors. Additional interviews were conducted for both nondonors and lapsed donors to validate and expand the already identified patterns, concepts and themes [49]. The coded data were compared and discussed among the first three authors in routine research meetings and were discussed with and agreed upon by the fourth author, who was a department operations manager and a registered nurse of the BTS during the study period. Selected interview quotes were translated from Chinese to English, and back‐translation from English to Chinese was then conducted to confirm the accuracy of the translations.

### 2.5 Study Rigour and Data Trustworthiness

To ensure the rigour of the study design and methods, the criteria developed by Lincoln and Guba [50] were considered. Data collection and analysis were conducted in accordance with the Consolidated Criteria for Reporting Qualitative Research (Supporting Information: Appendix 2) [51]. The interviewed participants were asked to read their transcripts to ensure that their meanings were accurate, and all the participants confirmed the content of their transcripts. Coding meetings were held monthly among the first three authors to ensure agreement. The fourth author, as a department operations manager of the BTS, regularly provided opinions on the coding procedure in these meetings. Consensus was achieved among all four authors.

## 3 Findings

Participant demographics are shown in Tables 1 and 2. There were 40 lapsed donors and 40 nondonors in this study. The majority of the nondonor participants were in the younger age group of 19 to 30 years of age, having received university education or above. The majority of the lapsed donor participants were in the elder age group of 51–60 years of age, having received postsecondary or university education. Eleven participants had donated blood at least 6 times before lapsing. Nondonor and lapsed‐donor participants alike reported multiple reasons for not donating blood during the COVID‐19 pandemic. These factors were not related to the pandemic alone, instead, nondonation was sometimes anchored in prepandemic perceptions and experiences arisen before the outbreak.

**Table 1:** A summary of participant distribution.

**Nondonors (ND) (ND001–ND040)**	
**Sex**	
Woman	26
Man	14
**Age**	
19–30	26
31–40	4
41–50	1
51–60	5
61–65	4
**Education level**	
University or above	32
Postsecondary	2
Secondary	5
Primary	1
**Lapsed donors (LD) (LD001–LD040)**	
**Sex**	
Woman	29
Man	11
**Age**	
19–30	5
31–40	3
41–50	9
51–60	16
61–65	7
**Education level**	
University or above	22
Postsecondary	11
Secondary	7
Primary	0
**Times of donation before lapsing**	
1–5	29
6–10	6
11 times or above	5

**Table 2:** Demographics of individual participants.

Informant ID	Gender	Age	Education level	Occupation	Times of donation
**Nondonors (ND)**					
ND001	Woman	28	University	Freelancer	0
ND002	Woman	28	University	Marketing Assistant Manager	0
ND003	Woman	26	Master's	Research Assistant	0
ND004	Man	25	University	Student	0
ND005	Man	23	University	Student	0
ND006	Woman	22	University	Website Designer	0
ND007	Woman	32	University	Administration in nongovernmental organization	0
ND008	Woman	20	University	Student	0
ND009	Woman	22	University	Student	0
ND010	Woman	22	University	Student	0
ND011	Woman	24	University	Administration in nongovernmental organization	0
ND012	Man	24	Master's	Research Assistant	0
ND013	Man	26	University	Chinese Medicine Practitioner	0
ND014	Man	26	University	Research Assistant	0
ND015	Man	24	University	Unemployed	0
ND016	Man	23	University	Engineer	0
ND017	Man	22	University	Student	0
ND018	Man	24	University	Student	0
ND019	Woman	27	Doctorate	Student	0
ND020	Woman	27	University	Researcher	0
ND021	Woman	60	Secondary	Retiree	0
ND022	Man	19	Associate Degree	Student	0
ND023	Man	48	University	Salesperson	0
ND024	Woman	26	University	Occupational Therapist	0
ND025	Woman	31	University	Flight Attendant	0
ND026	Woman	52	Secondary	Housewife	0
ND027	Woman	60	Master's	Retiree	0
ND028	Woman	64	Primary	Retiree	0
ND029	Man	64	Higher Diploma	Retiree	0
ND030	Woman	25	Master's	Officer	0
ND031	Woman	55	University	Executive	0
ND032	Woman	53	Secondary	Part‐time Clerk	0
ND033	Man	65	University	Retiree	0
ND034	Woman	29	University	Arts Administration	0
ND035	Man	31	University	Retailing	0
ND036	Woman	24	Master's	Social Welfare	0
ND037	Woman	25	Master's	Freelance Copywriter	0
ND038	Woman	34	Secondary	Tutor	0
ND039	Woman	64	Secondary	Clerk	0
ND040	Woman	29	Tertiary	Clerk	0
**Lapsed donors (LD)**					
LD001	Woman	24	University	Social Worker	1
LD002	Woman	26	University	English Tutor	~3
LD003	Man	23	Tertiary	Student	1
LD004	Woman	58	Higher Diploma	Retiree	1
LD005	Man	45	University	Accountant	3
LD006	Woman	55	Master's	Unemployed	1
LD007	Woman	58	Master's	Retiree	< 10
LD008	Woman	58	Tertiary	Housewife	1
LD009	Woman	60	University	Retiree	3
LD010	Man	39	Doctorate	Executive in university	1
LD011	Woman	26	University	Human Resources	1
LD012	Woman	26	University	Public Organization	1
LD013	Man	42	University	Education	2
LD014	Woman	62	Tertiary	Retiree	9
LD015	Woman	60	Secondary	Retiree	~33
LD016	Man	52	University	Businessman	2
LD017	Man	63	University	Retiree	1
LD018	Woman	43	University	Clerk	1
LD019	Woman	49	University	Unemployed	1
LD020	Man	57	Secondary	Civil Servant	~10
LD021	Man	63	University	Retiree	1
LD022	Woman	62	Master's	Retiree	2
LD023	Man	52	Secondary	Interior Design	1
LD024	Woman	50	Tertiary	Clerk	~4
LD025	Woman	52	University	Social Worker	~10
LD026	Woman	64	Tertiary	Retiree	1
LD027	Woman	60	Tertiary	Exercise Instructor	10
LD028	Woman	41	Tertiary	Housewife	2
LD029	Woman	64	Master's	Retiree	15–20
LD030	Woman	55	Tertiary	Clerk	1
LD031	Woman	54	Tertiary	Retiree	1
LD032	Woman	45	University	Housewife	3
LD033	Man	36	University	Civil Servant	1
LD034	Woman	48	University	Housewife	> 20
LD035	Woman	65	Secondary	Housewife	1
LD036	Woman	57	Secondary	Clerk	1
LD037	Woman	58	Tertiary	Unemployed	10
LD038	Woman	32	University	Flight Attendant	1
LD039	Woman	47	Secondary	Housewife	~30
LD040	Man	58	Secondary	Retiree	> 20

As noted in Section 2.4, themes from these interviews were assigned to the most appropriate social level from the CMA framework. We have structured our data reporting by those levels and do so in the order in which interviewees tended to mention the themes. While this may not be the most conceptually straight‐forward approach, it is intended to help readers better understand the priority interviewees accorded various barriers, and thereby illustrate their thought processes. In the interview quotes cited in the following sections, ND indicates nondonors, whereas LD indicates lapsed donors. In the ND group, one informant reported having been to a donor centre intending to make the first donation but was rejected after screening.

### 3.1 Theme 1: Barriers to Blood Donation During the COVID‐19 Pandemic

The barriers discussed in this theme are pandemic‐related and are started at the CMA's intermediate‐social level, which the participants mentioned as having the most proximate and fundamental effects on their perceptions and behaviours at other levels.

#### 3.1.1 Intermediate‐Social Level: Quarantine Policy

The intermediate‐social level concerns the barriers in relation to institutional policies. All participants mentioned the government's quarantine and social distancing policies as the most proximate and potent factor of not donating:

*I hesitate to donate blood at this time because it requires close contact with the staff and other donors in the donor center. The government has always said it is very important to keep social distance from others…. If anyone is infected, then we will all have to be quarantined in Penny's Bay* [quarantine center] *and receive compulsory virus testing*.

(LD014)

*The government is asking us to stay at home and avoid going out unless necessary. I don't think the government would encourage people to go to donate blood now. My family and I will be quarantined in Penny's Bay* [quarantine center] *if I get infected*.

(ND001)

#### 3.1.2 Individual Level

The individual level concerns the barriers in relation to personal and individual perceptions and experiences, as well as the influence of one's social support network. In many cases, interviewees' perceptions about blood donation during the pandemic were affected by the previously mentioned institutional policies at the intermediate‐social level.

##### 3.1.2.1 Blood Donation as an Unnecessary Activity

All participants maintained only the activities that they perceived as necessary. Their definitions of ‘necessary’ and ‘unnecessary’ activities were shaped by the government's infection control policies at the intermediate‐social level:

*The government has never said blood donation is necessary during COVID, so blood donation is not a necessity…. Blood donation is additional and optional during COVID, so I would avoid it*.

(ND024)

Although nearly half of the lapsed donors were aware of the necessity of blood donation during the pandemic, the government's quarantine and social distancing policies had overridden their sense of the necessity of making blood donations:

*I understand many patients would still need blood regardless of whether there is COVID or not…. However, during COVID, you can only maintain things that are really necessary because the government has always been emphasizing going out only if necessary. Giving blood is not necessary during COVID because the government has never said giving blood is necessary*.

(LD012)

##### 3.1.2.2 Blood Donation as Risky Crowd Gathering

Blood donation was perceived as dangerous during the pandemic, in part because almost all participants perceived this activity as involving the gathering of crowds because of the physical setting of donor centres that would expose them to a higher risk of infection. This perception was also affected by the intermediate‐social level as the government had been asking people to avoid crowd gatherings during the pandemic:

*The environment [of donor centers] makes me worry about whether I can get infected during the donation. I can never know if the person sitting next to me carries the virus. The donation area is not separated into individual rooms, and the donation seats are very close to each other. I feel very unsafe about such a crowded environment*.

(LD037)

*Blood donation is risky because it involves the gathering of people. The government has said we should avoid gatherings…. I am worried about the environment because it is a gathering of people. I have never been to a donor center, but I can expect that I would not be the only one to donate. It is very difficult to ensure that the place is virus‐free because there are people gathering in the center*.

(ND004)

##### 3.1.2.3 Higher Perceived Risk of Blood Contact

More than half of the lapsed‐donor and nondonor participants perceived blood as an agent of COVID‐19 transmission and perceived donation as a particularly risky invasive procedure involving blood:

*Blood is dangerous because many infectious diseases can be transmitted through it, and I think this pneumonia* [COVID‐19] *is no exception. If the blood and the donation procedure are not handled well, others can become infected…. Blood donation is invasive, and you will have a wound through which you can easily become infected*.

(LD003)

*Many infectious diseases are transmitted through blood. As COVID is an infectious disease, I think it can be transmitted through blood. If COVID can be transmitted through a person's droplets, then of course it can be transmitted through blood because blood should have more of the virus than droplets do, right?*

(ND010)

#### 3.1.3 Micro‐Social Level

In this study, micro‐social level factors, that is, interaction between healthcare personnel and patients, involved how nondonors and lapsed donors perceived imagined interaction with BTS personnel and sites.

##### 3.1.3.1 Stigma About Healthcare Personnel

More than half of the participants perceived BTS personnel as a generally risky group during the pandemic because they were associated with healthcare personnel, viruses, bacteria and patients. Participants often referred BTS personnel as healthcare personnel. As indicated by the phrasing and content of this lapsed donor's comment, BTS personnel were conflated with healthcare personnel who contact patients, even though BTS personnel do not actually provide medical care:

*Healthcare personnel are a risky group…. They will leave the virus and contaminate things because they contact patients. It would be safer for me to have less contact with them*.

(LD016)

Such conflation had also made some nondonors perceive BTS personnel as dirty during the pandemic:

*Healthcare personnel are dirty because their work environment is full of viruses and bacteria, and they have to see sick people. Although they have protective gowns and materials, it is still not guaranteed that they can be virus‐free. If you are sick, then you have no choice but to see them. But if you are not sick, there is no reason for me to bear the risk just for blood donation*.

(ND016)

##### 3.1.3.2 Stigma About Medical‐Related Sites

Participants' perceptions of BTS personnel were closely correlated with the perception of medical‐related sites as hazardous during the pandemic. Although the participants understood well that donor centres were not hospitals, the perception of danger persisted, which affected how they viewed the personnel working there:

*I know donor centers are not hospitals, but they are associated with hospitals, so the centers and their staff would not be safe anyway…. I am afraid of going to a donor center at this time because if I donate blood, a nurse would need to touch me, and I would be at risk of getting infected…. Anyway, I avoid going to doctors and dentists at this time because donor centers and clinics, like hospitals, are dangerous*.

(LD031)

Such perceptions of medical‐related sites reinforced the mental association between blood donation and risk of infection, thereby discouraging participants from donating:

*My feeling is that donor centers and hospitals are closely related, and hospitals make me think of viruses and bacteria. I know that sounds irrational, but it really makes me feel that donating blood at this time is unsafe*.

(ND007)

##### 3.1.3.3 Fear of Infection Due to Error by BTS Personnel

The blood donation process involves BTS personnel, and according to the participants, this ostensibly suggests that human error would be impossible to avoid completely. This perception served as a barrier for nearly half of the participants to donating blood during the pandemic:

*I worried that the staff…may be too busy and forget some steps [in the procedure]. They are human, not robots; you cannot expect them to achieve zero mistakes. However, if they cannot achieve zero mistakes, then I will be subject to greater risk of getting infected*.

(LD026)

This perception was more prevalent among nondonors:

*Anything that involves humans can mean a potential risk, and you may have higher risk of getting infected if a person is not cautious about infection prevention. You are unable to donate blood through technology with no human involved at this moment. If blood donation could be done through a robot or AI [artificial intelligence], I would feel safer; a robot or AI would not make any mistake, but a human would*.

(ND023)

#### 3.1.4 Macro‐Social Level

##### 3.1.4.1 Ethnomedically Informed Perception of Blood Donation's Impact on Immunity

The macro‐social level concerned barriers engendered by pandemic‐related ethnocultural health beliefs, social norms and ideology. Due to Chinese ethnomedical belief that blood loss weakens immunological resistance to infections, more than half of the participants believed that blood donation would increase their vulnerability to infection:

*Donating blood means losing blood, and you will be weaker. You may have a higher chance of becoming infected because you will become weak after losing blood*.

(ND030)

This perception was more prevalent among participants who had lapsed from blood donation. Their past experiences with blood donation had reinforced their belief in weaker immunity after donating blood, which discouraged them from donating during the pandemic:

*I would feel my body and energy become weaker after donating blood. It is a feeling where I would get dizzy and tired for a few days after donation…. Therefore, I believe that losing blood can lead to weaker immunity. I don't think it is good to donate blood during COVID because you should save your blood and immunity*.

(LD022)

##### 3.1.4.2 Collective Responsibility to Prevent Infection (e.g., Protecting Neighbours/Social Network and Blood Recipients)

The government's COVID‐19 infection control and quarantine policy emphasised a sense of collective responsibility, in that one's potential infection does not merely affect oneself, but also others (such as close contacts). This resulted in the construction of new social norms and ideology at the macro‐social level. This was reported as a barrier to blood donation by all the participants:

*I imagine that my family would not support me donating blood at this time. If I get infected, they may blame me for making them go to the quarantine camp. I am afraid of becoming infected not because I am afraid of death, but because it is no longer an individual matter; you can cause trouble for others who are required to undergo quarantine*.

(LD017)

*If I become infected from blood donation, I will affect my family members and my colleagues because they will be sent to the quarantine camp…. If I become infected from my workplace, that's fine because work is a necessity. However, if I become infected from blood donation, those who are affected by me will definitely blame me because blood donation is unnecessary at this time*.

(ND039)

Collective responsibility, thus, was perceived to be correlated with the society's belief of whether a behaviour is ‘necessary’, defined by infection control policy at the intermediate‐social level. Collective responsibility was also interpreted as ‘not burdening healthcare personnel’ according to the participants' perceptions. This also served as a noteworthy barrier to the participants:

*There is no urgency to donate blood. Avoiding getting infected is a much higher priority because I do not want to increase the burden and workload of healthcare personnel. If I go to donate blood, yes, I can help patients; but if I get infected, I also burden the healthcare personnel*.

(LD005)

The sense of concern about potentially transmitting COVID‐19 to recipients of blood also demotivated more than half of the lapsed and nondonor participants from engaging in blood donation:

*It is not a good time to donate blood because you can never guarantee that you are virus free. If you are carrying the virus and donating blood, then you are actually not helping the recipient; you may make them even more ill…. You can say you have to do a virus test before the donation, but the pneumonia* [COVID‐19] *has an incubation period, so how can you be sure* [you are virus free]*? Those who need to receive blood are very sick already; if my blood carries the virus, will I make the patient die? Will I make a negative effect on the patient's life? I could not forgive myself if this came to pass*.

(ND018)

*I donate because I want to help people. However, you can never know whether you are really helping people by donating blood in this pandemic. If I did not know I am infected and I go to donate, I may kill the patient who receives my blood*.

(LD032)

### 3.2 Theme 2: Underlying Barriers to Blood Donation Preceded the Pandemic

As mentioned, the reasons participants did not donate blood during the COVID‐19 pandemic were not merely due to the pandemic itself, but also embedded in their perceptions and past experiences of blood donation before the pandemic. Prepandemic deterrents to donation spanned the levels of the CMA framework.

#### 3.2.1 Individual Level: Feeling Frustrated From Deferral

Being deferred for blood donation was an experience for more than half of the lapsed donor participants before the pandemic. In most cases, being deferred was not a pleasant experience and negatively affected their motivation to donate in the future:

*I still wanted to donate blood 5 or 6 years ago. Yet I was told that my hemoglobin was too low for three attempts, so I gave it up. It is not a happy thing to be rejected. You need to plan ahead when you decide to donate, and you also need to adjust your schedule to suit the opening hours of donor centers. It is very frustrating when you are rejected*.

(LD035)

#### 3.2.2 Micro‐Social Level: Unpleasant Experience With the BTS Personnel at Donor Centers

For nearly half of the lapsed donor participants, unpleasant interactions with BTS personnel during prior donations deterred them from returning for blood donation. Lapsed donors cited poor staff attitude and a perceived lack of skill as key to their dissatisfaction:

*Last time when I donated blood, a male nurse was very impatient…. He stuck the needle into my arm many times but couldn't get my blood. He kept telling me to relax…. However, he was very impatient, and I got even more nervous from his impatience. I could feel that he just wanted to get the job done as soon as possible, and he became more and more impatient after he had failed so many times. He did not realize that his impatience could make me become more nervous…. This experience was very discouraging, and I wouldn't want to donate anymore*.

(LD002)

#### 3.2.3 Intermediate‐Social Level: Perceived Discriminatory Donor‐Screening Policy

In Hong Kong, BTS requires all potential donors to undergo a screening process before blood donation. The screening involves a self‐administered questionnaire, as well as history taking and physical screening by nurses in the donor centres. However, some participants, including some who identified as homosexual, indicated that the screening process was discriminatory to them:

*As a homosexual, I feel rather disturbed in the screening because the questionnaire has a homophobic question asking whether you are a gay or not…. I think it is a kind of discrimination targeting gays because they only ask if you have any sex with a man if you are a man…. The whole thing was so confrontational, and I do not donate anymore*.

(LD010)

Another participant, who identified as heterosexual, shared a similar viewpoint:

*The questionnaire would ask you if you're a sex worker, a drug addict, or a gay. I think it's discriminating against these people…. A person with any of these attributes doesn't necessarily mean that their blood would be dirty. You cannot label them. Even if you're a heterosexual, you may have AIDS anyway. I don't feel comfortable with this because it appears to be discriminatory, and I do not want to donate again*.

(LD020)

#### 3.2.4 Macro‐Social Level: Political Economy of Blood Donation

The macro‐social level of the CMA framework indicates that the global political economy can affect people's health perceptions and behaviour. This is evident in the realm of blood donation in Hong Kong. Hong Kong and mainland China have a difficult political relationship, and this was connected to suspicions about who really benefits from donations. More than half the participants reported being demotivated by this, as in the following participant's perception:

*The whole process isn't transparent, and you can never know how many bags of blood that every hospital receives every day. They* [BTS] *won't tell how much blood they obtain every day, and I have no idea where the donated blood goes…. It is possible that they just dump the blood into the sea, or they may take our blood for DNA tests. They may sell our blood to the mainland or to other countries for money. After all, blood can be sold for money on the mainland, so I think it's really possible for the Red Cross to sell the blood there*.

(LD001)

Another participant hesitated to donate blood because she perceived that the blood donation system has attracted an additional burden from mainland China into the healthcare system of Hong Kong:

*After the handover, more and more people from the mainland have come to Hong Kong to use our healthcare system. My friends who are nurses told me that many patients are from the mainland, especially those who need a blood transfusion. Therefore, I think our blood inventory attracts many people from the mainland and adds a burden to our healthcare…. If I donate, I will be like a gear wheel supporting these mainlanders continuing to come to Hong Kong for free blood*.

(ND011)

## 4 Discussion

### 4.1 Participants' Nondonation as a Product of Interaction of the Four Social Levels of the CMA Framework

**Figure 2:**
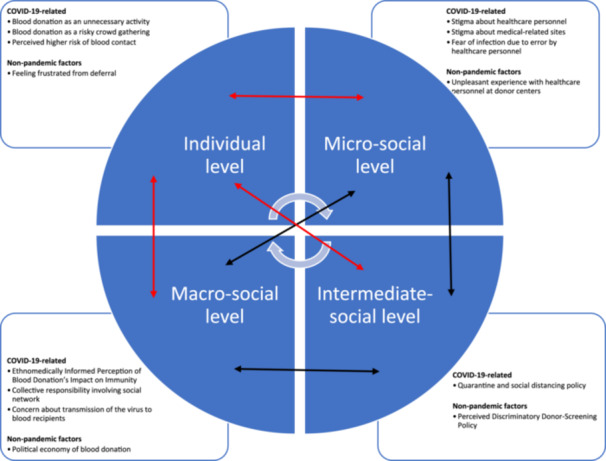
A concept map of the participants' non‐blood‐donation during the COVID‐19 pandemic following the critical medical anthropology framework [19], where red arrows denote participants' dissatisfaction and hope [33].

The adopted CMA framework has shown that the participants' experiences are a product of interaction among people's actions at both individual and microsocial levels, policies at the intermediate‐social level and political and economic forces at the macro‐social level. The decision not to donate during the pandemic cannot be explained by pandemic factors alone. Although the participants' sense of being a ‘good citizen’ arising from the new social norms developed in the pandemic at the intermediate level (quarantine policy) and the macro‐level social structure (collective responsibility) had affected their micro‐level perceptions (blood donation as unnecessary and risky and healthcare personnel as dangerous), their experiences at different social levels preceded the pandemic had played an important embedding role in reinforcing their nondonation during the pandemic (Figure 2) [19]. Simultaneously, the microspheres reinforced the macrosocial structures.

The government's quarantine and infection control policies at the intermediate‐social level are the most proximate factors for the participants not donating. Interviewees interpreted these policies as indicating that donating blood was an unsafe, unnecessary and nonurgent activity that involved risky crowd gathering. In particular, the environment of donor centres was demotivating, which is consistent with previous studies showing the concerns about close contact with other blood donors dissuaded people from donating blood during the pandemic [52]. Blood donation conflicted with the participants' understandings of institutional appeals for infection control. As noted in past studies, those with a stronger sense of adherence to the COVID‐19 infection‐control guidelines are less likely to donate blood [53]. Indeed, all the participants perceived adherence to the infection‐control guidelines as a collective responsibility, which significantly prevented them from donating blood in the COVID‐19 pandemic. Such a sense of collective responsibility made the participants believe that blood donation during the pandemic would not be supported by their social network, and peer pressure and the acceptance of others have been crucial for people donating blood during the COVID‐19 pandemic [54]. Quarantine policies at the intermediate‐social level affected macro‐social ideology and then the participants' perceptions and behaviours at the individual level. Under the established policies, the prevention of infection was socially constructed as a collective responsibility, which had become a social and cultural norm at the macrosocial level during the pandemic. A study in Germany notes that personal moral norms of donation had become even more ineffective for nondonors during the pandemic when the personal moral norms had become focused on infection reduction [16]. Without the government's appeal about the importance of blood donation during the pandemic, social and cultural macrosocial norms made infection prevention become the most prioritised. This overrode the importance of blood donation and demotivated the participants from donating blood as a result.

The stigma about healthcare personnel and facilities as being contaminated served as a notable barrier for the participants at the micro‐social level. The participants stereotyped BTS personnel as a risky and dangerous group during the pandemic. To the participants, blood donation and donor centres were associated with hospitals, viruses, bacteria and COVID‐19. Such preconceptions have also been documented in other parts of Asia as a demotivating factor for people who might ordinarily donate blood [55]. Besides, blood was perceived as dangerous by participants at the individual level, as many of them extrapolated the blood‐borne transmissibility of other infectious diseases to COVID‐19. This knowledge discouraged them from donating during the pandemic, which is similar to the situation in other Chinese communities [3]. This perception at the individual level also reinforced the stigma about BTS personnel in donor centres at the micro‐social level because they are required to be involved with blood in the donation procedure. People can be culturally regarded as dirty or dangerous when they are in contact with ‘polluted’ and ‘contaminated’ things, which can result in their seclusion from society [56]. These notions strongly discouraged the participants from donating blood during the pandemic.

The nondonation decision of the participants during the pandemic cannot be explained by pandemic factors at four social levels alone. Their experiences at different social levels preceded the pandemic also embedded in reinforcing their nondonation decision during the pandemic. Some studies show that people protest against the new social norms (‘the new normal’) initiated by governments during the COVID‐19 pandemic [57], but the present participants complied with the government's pandemic policy. Nevertheless, their nondonation showed hints of resistance against the structural issues in blood‐donation practice in Hong Kong that had been embedded in the time before the pandemic. These pandemic‐preceded factors are discussed further below.

### 4.2 Participants' Nondonation as an Expression of Dissatisfaction Embedded in the Time Before the Pandemic

Not engaging in desirable health behaviour has been suggested as a form of advocacy calling for change in health structures, delivery of healthcare, and access to social programs and medical technology [34]. Echoing the recent CMA appeal by Dutta and Basu [58], the decision not to donate blood could show a hint about participants' expression of dissatisfaction toward the blood donation institution and policy, and such dissatisfaction has often been embedded in the participants' minds before the pandemic. Nondonation during the pandemic, we argue, should not be viewed as isolated incidents that only happened in the pandemic, but nondonation has a processual nature in which these demotivating factors have been occurring over time before the pandemic and they influence each other.

Many participants, especially those who had lapsed from blood donation, expressed dissatisfaction toward deferral, BTS personnel and donor screening policy. Participants commonly expressed frustration with deferral and BTS personnel. This should be noted as an important embedding factor as past literature has also demonstrated that deferral [59] and unsupportive donor centre staff [60] can be significant barriers for donors to return for blood donation. Some participants were also dissatisfied with the current donor screening policy, which excludes people with nonmainstream sexual orientation.

In the context of the difficult political relationship between Hong Kong and mainland China at the macrosocial level, our study shows that the political economic situation of blood donation had also demotivated the participants from donating blood before the pandemic. The potential monetary value of blood in mainland China was widely perceived by the participants, which undermined their trust in the BTS and served as a prevalent factor in their nondonation even before the pandemic. The participants' decision not to donate is consistent with past literature [29, 60], which showed that trust in a healthcare system is a significant factor that can affect people's willingness to donate blood. The participants' nondonation can show hints of their dissatisfaction with the political and economic structure of Hong Kong and of their hope for more transparent blood donation policy.

### 4.3 Comparing Barriers to Donation Reported by Non‐ and Lapsed Donors

To both the non‐ and lapsed donor participants, their immediate barriers to donating were pandemic‐related. Quarantine policy made both groups of participants perceive the behaviour of blood donation as unnecessary and risky that could impose a negative impact on others. However, lapsed donors tended to have a higher sense of blood donation as important during the pandemic than the nondonors. The pandemic also affected both groups of participants to view healthcare personnel negatively. As BTS personnel were often perceived as healthcare personnel who provided patient care by the participants, this had demotivated them to donate blood during the pandemic. Another alarming finding noticed by both the lapsed‐ and non‐donors is their common perception of blood as the transmission route of COVID‐19, which has implications for the blood donation appeal in future pandemics.

Prepandemic barriers affecting the two groups of participants not donating were noted. Lapsed donor participants tended to report more about their unpleasant experiences with the past donation as part of their reasons for lapsing. These unpleasant experiences could be related to deferral, staff of donor centres and donor screening policy. The uncomfortable physical feeling after donation also made lapsed donors to have more hesitation in donating during the pandemic. Nondonor participants also reported barriers prepandemic, but these prepandemic barriers were more due to their suspicion of the blood donation system, which was originated from the macro‐level political hardship between Hong Kong and mainland China.

## 5 Limitations

The findings should be interpreted with caution due to the highly educated sample. In qualitative methodologies, the selection of a theoretical framework to guide research ideally entails a commitment to research methods that harmonise with the underlying theoretical assumptions and demands. We acknowledge that the use of the CMA framework would suggest the value of multiple data collection methods—in particular, participant observation, which holds a privileged place in the anthropological toolkit. However, because infection control policies implemented during the study period had made participant observation impractical or infeasible, the findings are based on a substantial reliance on the interview data. Given our aim of remaining analytically ‘close to’ participant perspectives and words, as required by the qualitative descriptive approach, we opted to conduct individual semi‐structured interviews. Despite this limitation, using CMA as the analysis framework provides a holistic and comprehensive analysis on the concerns of nondonor and lapsed donor participants in blood donation during the pandemic, and posits how individual perceptions, collective meanings and structural constraints interact to explain these concerns. Our study could still provide a contextualised understanding for transferability of the findings, providing an argument for engaging in future work that is designed from a CMA perspective.

## 6 Conclusion

Following the CMA framework, we suggest that the direction for the promotion of blood donation in future epidemics should not merely focus on removing pandemic‐related barriers. The underlying reasons and experiences that arose before the pandemic are notably responsible for people's decisions not to donate during the pandemic. Addressing only the barriers arising from the pandemic may not be adequate, and it would be beneficial to address these prepandemic factors to enhance the motivation for blood donation among nondonors and lapsed donors.

## Author Contributions

**Judy Yuen‐man Siu:** conceptualisation, data curation, formal analysis, funding acquisition, investigation, methodology, project administration, resources, supervision, validation, visualisation, roles/writing – original draft, writing – review and editing. **Engle Angela Chan:** conceptualisation, data curation, formal analysis, funding acquisition, investigation, methodology, supervision, writing – review and editing. **Angus Siu‐cheong Li:** data curation, formal analysis, investigation, project administration, roles/writing – original draft, writing – review and editing. **Yik Mun Lee:** funding acquisition, resources, writing – review and editing. All the authors have read and approved the final manuscript.

## Ethics Statement

Ethics approval was obtained from the Human Subjects Ethics Subcommittee of The Hong Kong Polytechnic University before the start of the study (HSEARS20171013002). Participants' written informed consent (for face‐to‐face interviews) and audio‐recorded informed consent (for online interviews) was obtained before data collection.

## Consent

All participants provided written consent for taking part in this study.

## Conflicts of Interest

The authors declare no conflicts of interest.

## Supporting information

Supporting information.

Supporting information.

## Data Availability

The data that support the findings of this study are not publicly available due to the participant confidentiality and privacy but are available from the corresponding author upon reasonable request.
