# Supplementary material for: Barriers to Engaging in Blood Donation During the COVID‐19 Pandemic Among Nondonors and Lapsed Donors in a Chinese Community: A Critical Medical Anthropology Perspective
Source: Health Expect. 2025 Apr 2;28(2):e70236. doi: 10.1111/hex.70236 (PMC11965271; doi:10.1111/hex.70236)
Supplement: Supplementary file 1 — Supporting information. [file HEX-28-e70236-s001.docx]

**Appendix 1. Interview Guide for Nondonors and Lapsed Donors**

**Probing questions are not listed in this interview guide as they were asked in response to informants’ feedback.*

With nondonors (n=40)

1. What is the meaning of blood for you?
   1. How important do you think blood is?
2. What are the meanings of blood donation to you?
3. What do you think about the act of donating blood?
   1. How important it is for one to participate in blood donation?
4. Do any of your family members, friends, colleagues, classmates, etc. participate in blood donation?
   1. What do you think about their participation in blood donation?
   2. Does their participation make you more interested in/more likely or less motivated to donate blood?
5. Have you ever considered donating blood?
   1. How important is it for you to donate blood?
   2. Can you talk about the reasons you have (have not) considered donating blood?
6. What would motivate you to make your first blood donation?
7. Do you think your cultural and/or religious beliefs influence your thoughts about blood donation? If yes, how and in what ways?
8. Can COVID-19 affect your thoughts in blood donation? If yes, how and in what ways?
9. Are you familiar with any promotion strategies to encourage blood donation (advertisement, etc.)? Can you talk about any that you are familiar with?
10. Do any of these strategies make you feel more motivated or less motivated to give blood? In what ways?
11. What strategies do you think might work to increase your motivation in blood donation?
12. Have you ever engaged in other kinds of donation and/or charity behavior?
    1. If yes, what are they? Can you compare these other kinds of donation and/or charity behavior with blood donation?
13. Do you have any other thoughts or ideas about blood donation?

With lapsed donors (n=40)

1. What is the meaning of blood for you?
   1. How important do you think blood is?
2. What are the meanings of blood donation to you?
3. What do you think about the act of donating blood?
   1. How important it is for one to participate in blood donation?
4. Do any of your family members, friends, colleagues, classmates, etc. participate in blood donation?
   1. What do you think about their participation in blood donation?
   2. Does their participation make you more interested in/more likely or less motivated to donate blood?
5. Why did you have your first blood donation?
6. Can you share your happy and unhappy experiences about blood donation?
   1. How do you think these experiences can motivate and/or demotivate you to donate blood?
7. How do you feel about the blood donation centres and the blood donation process?
   1. Probe experience, environment, interaction with healthcare providers…
8. Why did you not keep up with blood donation?
9. Do you think your cultural and/or religious beliefs influence your thoughts about blood donation? If yes, how and in what ways?
10. Can COVID-19 affect your thoughts in blood donation? If yes, how and in what ways?
11. Are you familiar with any promotion strategies to encourage blood donation (advertisement, etc.)? Can you talk about any that you are familiar with?
    1. Do any of these strategies make you feel more motivated or less motivated to give blood? In what way?
12. What strategies do you think might work to increase your motivation in blood donation?
    1. Have you ever engaged in other kinds of donation and/or charity behavior? If yes, what are they? Can you compare these other kinds of donation and/or charity behavior with blood donation?
13. Do you have any other thoughts or ideas about blood donation?
