# Supplementary material for: Barriers to Engaging in Blood Donation During the COVID‐19 Pandemic Among Nondonors and Lapsed Donors in a Chinese Community: A Critical Medical Anthropology Perspective
Source: Health Expect. 2025 Apr 2;28(2):e70236. doi: 10.1111/hex.70236 (PMC11965271; doi:10.1111/hex.70236)
Supplement: Supplementary file 2 — Supporting information. [file HEX-28-e70236-s002.docx]

**Appendix 2. Cross-checking with the Consolidated Criteria for Reporting Qualitative Research (COREQ): 32-item checklist (Tong et al., 2007)**

| **Domain 1: Research team and reﬂexivity** | |
| --- | --- |
| Personal Characteristics | |
| 1. Interviewer/facilitator: Which author/s conducted the interview or focus group? | ASL as the interviewer for all the interviews conducted.  JYS and EAC as interview supervisors. |
| 2. Credentials: What were the researcher’s credentials? E.g. PhD, MD | JYS: Ph.D, M.Phil. B.B.Sc.  EAC: Ph.D, MA, B.Sc.N, RN  ASL: M.Phil., B.S.Sc.  YML: M.Sc.N, RN |
| 3. Occupation: What was their occupation at the time of the study? | JYS: Associate Professor.  EAC: Professor.  ASL: Research Associate.  YML: Department Operations Manager. |
| 4. Gender: Was the researcher male or female? | JYS: Female.  EAC: Female.  ASL: Male.  YML: Female. |
| 5. Experience and training: What experience or training did the researcher have? Relationship with participants | JYS: Anthropology, Public Health.  EAC: Nursing.  ASL: Sociology, cultural studies.  YML: Nursing.  All researchers do not have relationship with participants. |
| 6. Relationship established: Was a relationship established prior to study commencement? | The four researchers had no relationship established with the participants prior the study. |
| 7. Participant knowledge of the interviewer: What did the participants know about the researcher? e.g. personal goals, reasons for doing the research | The participants did not know about the interviewer prior the study. |
| 8. Interviewer characteristics: What characteristics were reported about the interviewer/facilitator? e.g. Bias, assumptions, reasons and interests in the research topic | The interviewer has two Masters in sociology and cultural studies. The interviewer was applying for a Ph.D programme at the time when he served as an interviewer in this study. Now he is a Ph.D candidate in criminology. He has no prior experience in blood donation research. |
| **Domain 2: Study design and theoretical framework** | |
| 9. Methodological orientation and Theory: What methodological orientation was stated to underpin the study? e.g. grounded theory, discourse analysis, ethnography, phenomenology, content analysis | Critical medical anthropology.  Qualitative descriptive research.  Thematic analysis. |
| 10. Participant selection Sampling: How were participants selected? e.g. purposive, convenience, consecutive, snowball | Purposive sampling.  Snowball sampling. |
| 11. Method of approach: How were participants approached? e.g. face-to-face, telephone, mail, email | Blood donation survey pool.  Posters in public facilities.  Social media (Facebook).  Participant referral. |
| 12. Sample size: How many participants were in the study? | 40 lapsed donors.  40 non-donors. |
| 13. Non-participation: How many people refused to participate or dropped out? Reasons? Setting | No participants refused to participate or dropped out from the interviews. |
| 14. Setting of data collection: Where was the data collected? e.g. home, clinic, workplace | Four interviews were conducted face to face in a private room of JYS’s institution.  Seventy-six interviews were conducted online. |
| 15. Presence of non-participants: Was anyone else present besides the participants and researchers? | No. |
| 16. Description of sample: What are the important characteristics of the sample? e.g. demographic data, date | The majority of the nondonor participants was in the younger age group of 19 to 30 years of age, having received university education or above. The majority of the lapsed donor participants was in the elder age group of 51 to 60 years of age, having received post-secondary or university education. Eleven participants had donated blood at least 6 times before lapsing. |
| Data collection | |
| 17. Interview guide: Were questions, prompts, guides provided by the authors? Was it pilot tested? | The interview question guide was developed with the continuous discussion and consensus among the four authors. The questions were developed basing on the past literature about blood-donation perceptions and barriers. The interview question guide was pilot-tested with those who shared the similar characteristics according to the sampling inclusion criteria to ensure the questions were comprehensible to the participants. Probing questions that are not in the interview guide were asked following the responses of each participant. |
| 18. Repeat interviews: Were repeat interviews carried out? If yes, how many? | No repeated interviews were conducted, but the participants were asked to read their interview transcripts after transcribed verbatim. |
| 19. Audio/visual recording: Did the researcher use audio or visual recording to collect the data? | Audio-recording was used for all interviews. |
| 20. Field notes: Were ﬁeld notes made during and/or after the interview or focus group? | The interviewer wrote down field notes and interview summary in an interview diary.  The first and second authors also noted the key points and impressions when listening to the audio recordings of all interviews. |
| 21. Duration: What was the duration of the interviews or focus group? | 1 to 1.5 hours for each interview. |
| 22. Data saturation: Was data saturation discussed? | Data saturation was achieved. |
| 23. Transcripts returned: Were transcripts returned to participants for comment and/or correction? | The participants were asked to read their interview transcripts after transcribed verbatim. They were asked if they found what they said in the interviews had been distorted. All participants agreed with their transcripts. |
| **Domain 3: Analysis and ﬁndings** | |
| 24. Data analysis: Number of data coders How many data coders coded the data? | The first three authors coded the interview data separately. Coded data were compared and discussed among the first three authors in routine research meetings and were discussed with and agreed upon by the fourth author. |
| 25. Description of the coding tree: Did authors provide a description of the coding tree? | The codes, categories, and themes deriving from the data, with supporting interview quotes, were documented in a coding table. |
| 26. Derivation of themes: Were themes identiﬁed in advance or derived from the data? | Identified codes, categories, and themes were consolidated into four social levels for analysis following the critical medical anthropology framework.  Other data not following this framework was also coded inductively and documented in the coding table. |
| 27. Software: What software, if applicable, was used to manage the data? | No software was used in data analysis. All the analyzed data and coding tables done by the first three authors were managed in the code book document in Microsoft Word. |
| 28. Participant checking: Did participants provide feedback on the ﬁndings? | Participants were invited to provide feedback on the verbatim transcripts of their interviews to ensure the interview transcripts had no distortion on their original meaning. |
| Reporting | |
| 29. Quotations presented: Were participant quotations presented to illustrate the themes / ﬁndings? Was each quotation identiﬁed? e.g. participant number | Participant quotations were presented with informant code in the report of findings. |
| 30. Data and ﬁndings consistent: Was there consistency between the data presented and the ﬁndings? | Consistency between the data presented and the ﬁndings was ensured. |
| 31. Clarity of major themes: Were major themes clearly presented in the ﬁndings? | Major themes were presented in the findings. |
| 32. Clarity of minor themes: Is there a description of diverse cases or discussion of minor themes? | Minor themes were reported in findings. |
